# Founding of the Ohio College of Dental Surgery

**Published:** 1882-04

**Authors:** A. Berry


### FOUNDING OF THE OHIO COLLEGE OF DENTAL SURGERY.

BY A. BERRY, D.D.S.

About four years ago the writer of this interviewed Dr. Rogers on the origin of the Ohio College of Dental Surgery. He said:

" Drs. Taylor and Cook came to my office and spoke to me on the subject of getting up a dental college. We talked the matter over, prepared a charter, and Dr. Cook went to Columbus and got a bill of incorporation through the lower house. When the time for it to come before the Senate approached, I went to Columbus to attend to it, and met Dr. Allen, who was there two days before me. The other dentists of the city had not been consulted in regard to the college, and they sent Dr. Allen to work against it. Their chief objection was that the charter did not provide for a board of trustees, and the college was to be controlled by the professors.

"Dr. Allen and I called on the chairman of the Senate Committee having charge of the bill, and he said, 'Gentlemen, agree between yourselves concerning the charter, and let me know.'

"Dr. Allen is a gentleman, and we had no difficulty in arranging the matter. We altered the charter to have a board of trustees, and I had the nominating of the first member ot it who would be its president, and named Dr. Aydelotte, a friend of mine."

"We called again on the chairman, who said to us: 'Gentlemen, you can go home; I will see that the bill passes.' It was passed without any objection."

A building, previously occupied for educational purposes, was rented, and the college opened in the autumn of 1845, with

JESSE W. COOK, M.D., D.D.S.,
Professor of Anatomy and Physiology.

JAMES TAYLOR, D.D.S.,
Professor of Practical Dentistry and Pharmacy.

MELANCTHON ROGERS, M.D., D.D.S.,
Professor of Dental Pathology and Therapeutics.

JESSE P. JUDKINS, M.D.,
Demonstrator of Anatomy.

The property where the college was commenced was subsequently purchased by the Ohio Dental College Association, and the present edifice erected.

